# Value of Contrast-Enhanced Ultrasound for Evaluation of Cervical Lymph Node Metastasis in Papillary Thyroid Carcinoma

**DOI:** 10.3389/fendo.2022.812475

**Published:** 2022-02-03

**Authors:** Fengkai Fang, Yi Gong, Liyan Liao, Fei Ye, Zhongkun Zuo, Xiaodu Li, Qi Zhang, Kui Tang, Yan Xu, Rongsen Zhang, Sijie Chen, Chengcheng Niu

**Affiliations:** ^1^ Department of Ultrasound Diagnosis, The Second Xiangya Hospital, Central South University, Changsha, China; ^2^ Department of Thyroid Surgery, The Second Xiangya Hospital, Central South University, Changsha, China; ^3^ Department of Pathology, The Second Xiangya Hospital, Central South University, Changsha, China

**Keywords:** contrast-enhanced ultrasound, conventional ultrasound, cervical lymph node metastasis, papillary thyroid carcinomas, benign cervical lymph nodes

## Abstract

The aim of the study was to evaluate the diagnostic value of contrast-enhanced ultrasound (CEUS) in distinguishing between benign and malignant cervical lymph nodes (LNs) in patients with papillary thyroid carcinoma (PTC). Two hundred and one cervical LNs (157 metastatic from PTC and 44 benign) were evaluated using conventional ultrasonography (US) and CEUS before biopsy or surgery. Histopathology was used as the gold standard. We evaluated the size, long axis/short axis ratio (L/S), fatty hilum, hyper-echogenicity, calcification, cystic change, peripheral vascularity and CEUS parameters for each lymph nodule. The CEUS parameters included enhancement type, homogeneity, perfusion type, ring enhancement, peak intensity (PI) index and area under the curve (AUC) index. Univariate analysis demonstrated that compared with benign LNs, malignant LNs more frequently had L/S < 2, absence of a fatty hilum, presence of hyper-echogenicity, presence of calcification, peripheral vascularity, hyper-enhancement, heterogeneous enhancement, centripetal perfusion, ring enhancement, PI index > 1 and AUC index > 1 on preoperative US and CEUS. Binary logistic regression analysis demonstrated that hyper-enhancement, centripetal perfusion, and ring enhancement are independent CEUS characteristics related to malignant LNs for their differentiation from benign LNs (all p < 0.05). Our study indicated that preoperative CEUS characteristics may serve as a useful tool to identify malignant cervical LNs from benign cervical LNs.

## Introduction

Papillary thyroid carcinoma (PTC) is the most common type of thyroid cancer with a significantly increased incidence in recent years, constituting up to 80% to 90% of all thyroid cancers (1, 2). Despite the indolent nature of PTC, cervical lymph node (LN) involvement is an important factor associated with local recurrence, and distant metastasis is quite common, which affects the surgical strategy, extent of surgery and prognosis (3, 4). Central and ipsilateral neck dissections when there is obvious nodal involvement from PTC might reduce the frequency of regional recurrence (1). Therefore, the accurate preoperative diagnosis and pretreatment evaluation of cervical LN metastasis is essential.

Preoperative neck ultrasonography (US) for cervical LNs is recommended to evaluate the surgical strategy and extent, especially for lymph node dissection according to the American Thyroid Association’s (ATA’s) guidelines (5). Metastatic LNs commonly appear with a round shape, the absence of an echogenic hilum, hyper-echogenicity, microcalcifications, cystic change and peripheral vascularity (5, 6). However, no single sonographic feature is adequately sensitive for the detection of LNs with metastatic thyroid cancer. The sensitivity of conventional US for central LNs is less than 50%, and it is approximately 70%-80% for lateral LNs (7, 8). Thus, it is urgently important to identify suspicious cervical LNs to allow for the design of individualized surgical treatment strategies to reduce the frequency of regional recurrence.

Contrast-enhanced ultrasound (CEUS) is a popular imaging modality with high sensitivity for vascularity and can provide qualitative and quantitative blood perfusion information compared with conventional US (9, 10). Studies have reported that CEUS can improve the specificity and sensitivity of US diagnoses to distinguish malignant LNs from benign LNs (11–13). However, to the best of our knowledge, the capability of CEUS, both qualitative and quantitative, for identifying metastatic cervical LNs from PTC has rarely been reported. Here, we studied the conventional US and CEUS features of metastatic cervical LNs in PTC patients and evaluated the added value of CEUS to conventional US characterization of metastatic cervical LNs from PTC.

## Methods and Materials

### Patients

The study was approved by the Ethical Committee of the Second Xiangya Hospital of Central South University in China and performed in accordance with the Declaration of Helsinki for human study. The requirement of informed consent from human subjects is sometimes waived by IRBs for protocols that include retrospective review of images acquired for clinical diagnostic purposes. From March 2021 to September 2021, 675 consecutive patients with thyroid nodules (containing at least one of the suspicious features) and abnormal cervical LNs who received conventional US and CEUS examinations were single-center prospectively enrolled in this study. The inclusion criteria were as follows: (i) a long-axis diameter of cervical LNs ≥ 0.5 cm and (ii) no invasive procedure such as LN gun biopsy or FNA, was previously performed. The following patients were excluded: (i) abnormal LNs without surgical pathologic results and (ii) patients with tuberculous nodes or other metastatic cervical LNs (not from PTC) confirmed by pathology. The final diagnosis of malignant cervical LNs was confirmed by histopathology after surgery. Meanwhile, LNs were considered benign if (i) the lymph node was diagnosed as benign on histology after surgery; (ii) the thyroid nodule was confirmed as benign on histology after surgery without lymph node dissection. For patients with multiple abnormal LNs, each lymph node was observed separately by multiple injections of contrast agent. Finally, 121 PTC patients with 201 abnormal cervical LNs (157 metastatic from PTC and 44 benign) were included in this study. In addition, laboratory examination results including serum free T4, free triiodothyronine (T3), TSH, thyroid peroxidase antibody (A-TPO) and thyroglobulin antibody (A-TG) were measured in all patients within 2 weeks of surgery.

### Conventional US and Color Doppler US

A Siemens Acuson S3000 US scanner (Siemens Medical Solutions, Mountain View, CA, USA) equipped with 9L4 (4–9 MHz) and 18L6 (6–18 MHz) linear array transducers was used for conventional US, and a 9L4 linear array transducer was used for CEUS. All examinations were performed by the same operator with fifteen years of experience in thyroid ultrasound diagnosis and five years of experience in performing CEUS to exclude bias from different operators and ensure optimized image quality. All selected LNs were evaluated by conventional B-mode US and color-Doppler US for the following US features: size (the largest diameter), long axis/short axis ratio (L/S ≥ 2 or < 2), fatty hilum (present or absent), hyper-echogenicity (present or absent), microcalcification (present or absent), cystic change (present or absent), and peripheral vascularity (vessels running along the capsule with or without centripetal branches, present or absent).

### CEUS and CEUS Analysis

CEUS was performed using contrast pulsed sequencing (CPS) technology (mechanical index, MI=0.07). SonoVue (Bracco, Italy) was injected intravenously as a bolus of 2.4 mL *via* a 20-gauge antecubital vein cannula, followed by a saline flush of 5 mL, with the timer started simultaneously. The LN imaging lasted at least 30 seconds. The CEUS videos were digitally recorded and analyzed with CEUS software (Contrast Dynamics, Mountain View, USA). The time-intensity curves (TICs) of LNs within selected regions of interest (ROIs) were acquired. Compared with adjacent tissue enhancement, the contrast enhancement features were classified as follows: enhancement type (hyper-enhancement, the enhancement intensity was as high as the internal jugular vein; iso-enhancement, the enhancement intensity was lower than the internal jugular vein, but higher than the surrounding adipose tissue; hypo-enhancement, the enhancement intensity was equal to or lower than the surrounding adipose tissue), enhancement homogeneity (heterogeneous or homogeneous), perfusion pattern (centripetal, perfusion of microbubbles from the periphery to the center of nodules or centrifugal, perfusion of microbubbles from the center to the periphery of nodules), ring enhancement (defined as a rim-like hyperenhancement around the lymph node, present or absent), peak intensity (PI, expressed as a percentage), and area under the curve (AUC, expressed in percentage by seconds). The PI and AUC of the nodules are reported as indices by the ratio of the ROI of LNs to the ROI of adjacent tissue.

### Reference Standard

The histopathological results after surgery were used as the only reference standard for the final diagnosis of malignant and benign LNs.

### Statistical Analysis

Statistical analysis was performed with SPSS version 21.0 software (SPSS, Chicago, IL, USA). Continuous data are presented as the mean and standard deviation (SD) and were compared by independent t test. Categorical data are presented as percentages and were analyzed by the Chi-square test. Binary logistic regression was used to assess significant US features and their independent association with malignant LNs. Statistically significant differences were determined with p < 0.05.

## Results

A total of 121 PTC patients with 201 cervical LNs (157 metastatic from PTC and 44 benign) were included in this study. The clinical characteristics of the patients are shown in **Table 1**. Among the 121 PTC patients, 79 patients had metastatic LNs from PTC and 42 patients did not have metastatic LNs (benign LNs) after LNs dissection. The average ages of PTC patients with and without metastatic LNs were 37.05 ± 11.86 years (range: 18-69 years) and 41.98 ± 11.53 years (range: 24-62 years), respectively (p =0.030), showing that the patients in this study with metastatic LNs tended to be younger. Ninety-three (76.9%) PTC patients were female, and 28 (23.1%) were male. Male patients constituted 27.8% of patients with metastatic LNs and 14.3% of patients without metastatic LNs (p = 0.092). Thirty-five (44.3%) patients with metastatic LNs had Hashimoto’ thyroiditis, and 20 (47.6%) patients without metastatic LNs had Hashimoto’ thyroiditis (p = 0.727).

**Table 1:** Clinical characteristics of the patients with malignant and benign lymph nodes.

Characteristics	Malignant LNs patients (n=79)	Benign LNs patients (n=42)	*P* Value
Age (y)	37.05 ± 11.86	41.98 ± 11.53	0.030*
Male sex	22 (27.8)	6 (14.3)	0.092
Hashimoto’ thyroiditis	35 (44.3)	20 (47.6)	0.727

*p < 0.05 was considered a significant difference.

**Figure 1:**
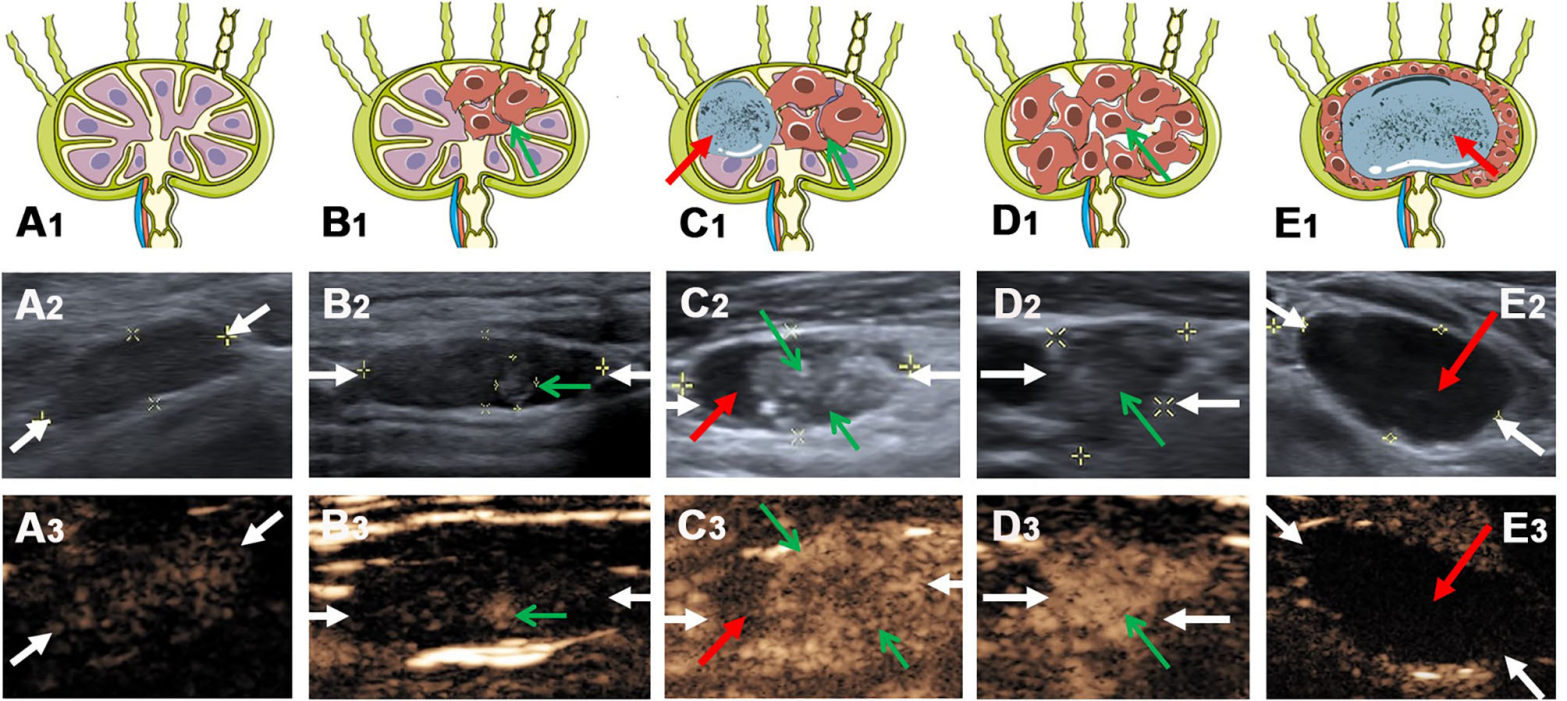
The typical metastatic LNs ultrasonographic performance on conventional US and CEUS. (A_1_) Schema of benign lymph node, (A_2_) reactive lymph node (white arrows) on conventional US and (A_3_) CEUS. (B_1_) Schema of local metastatic lymph node (green arrow indicate the metastatic lesion), (B_2_) local metastatic lymph node (white arrows) with a hyper-echoic appearance on conventional US and (B_3_) CEUS. (C_1_) Schema of metastatic lymph node with a hyper-echoic appearance, punctate echogenic foci and cystic change (green arrows indicate the metastatic lesion, red arrow indicates the cystic change), (C_2_) metastatic lymph node (white arrows) on conventional US and (C_3_) CEUS. (D_1_) Schema of metastatic lymph node (green arrows indicate the metastatic lesion), (D_2_) metastatic lymph node (white arrows) on conventional US and (D_3_) CEUS. (E_1_) Schema of metastatic lymph node with cystic change (red arrow indicates the cystic change), (E_2_) metastatic lymph node (white arrows) on conventional US and (E_3_) CEUS.

**Figure 2:**
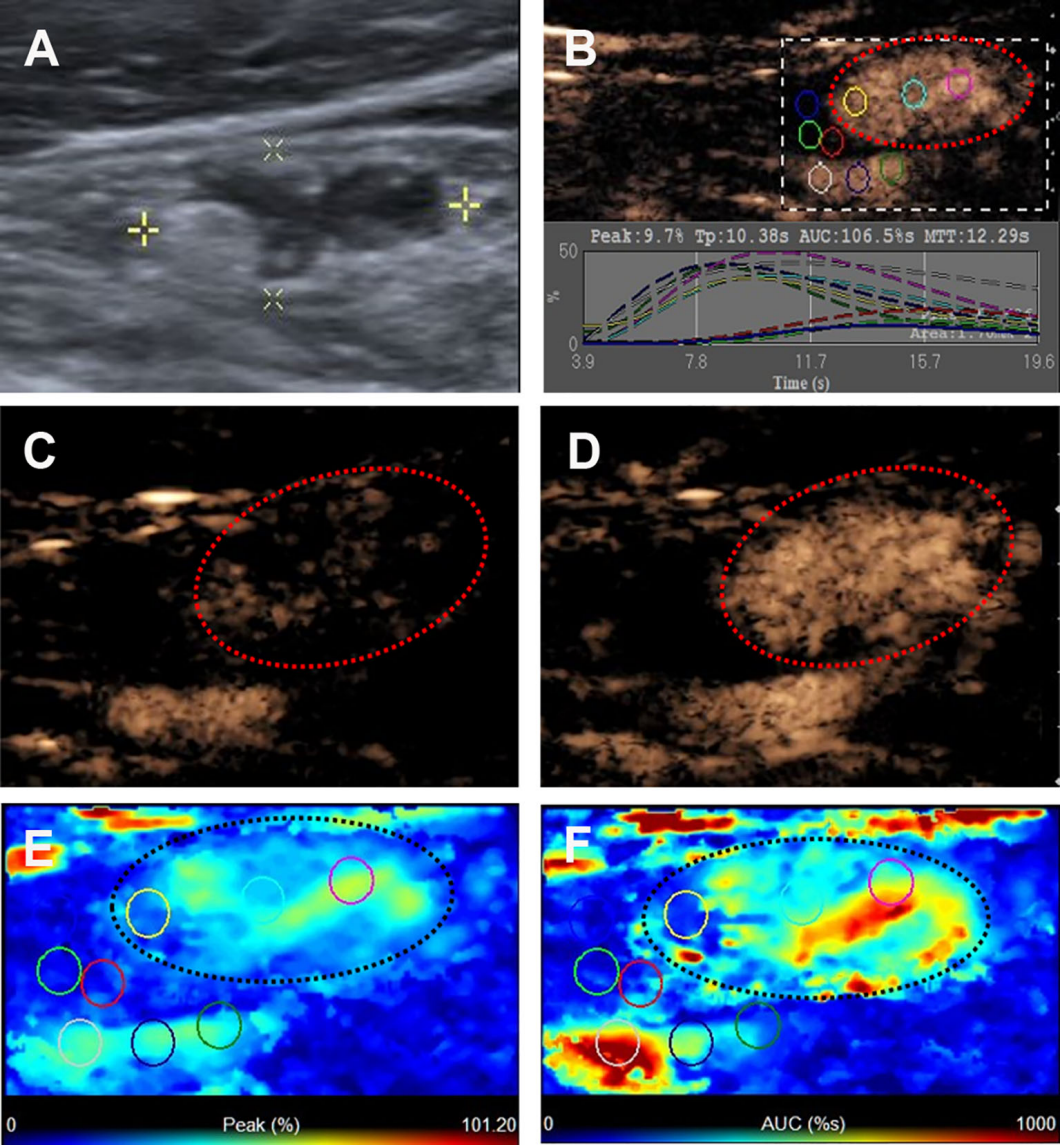
Metastatic lymph node in a 45-y-old female PTC patient. **(A)** The metastatic foci in a LN (white cross) identified on conventional US, showing the absence of fatty hilum, hyper-echoic appearance and punctate echogenic foci. **(B)** Numeric values of peak, time to peak, mean transit time and AUC were automatically calculated based on the time-intensity curve. **(C)** CEUS image 5 s after injection of contrast-enhanced agent, the LN enhanced from the periphery to the center. **(D)** CEUS image 11 s after injection of contrast-enhanced agent, the LN showed hyper-enhancement. **(E)** Parametric color map indicating the PI values for the center of LN was higher than that of the periphery of LN, equal to that of internal jugular vein and higher than that of the surrounding adipose tissue. **(F)** Parametric color map indicating the AUC values for the center of LN was higher than that of the periphery of LN, equal to that of internal jugular vein and higher than that of the surrounding adipose tissue.

**Figure 3:**
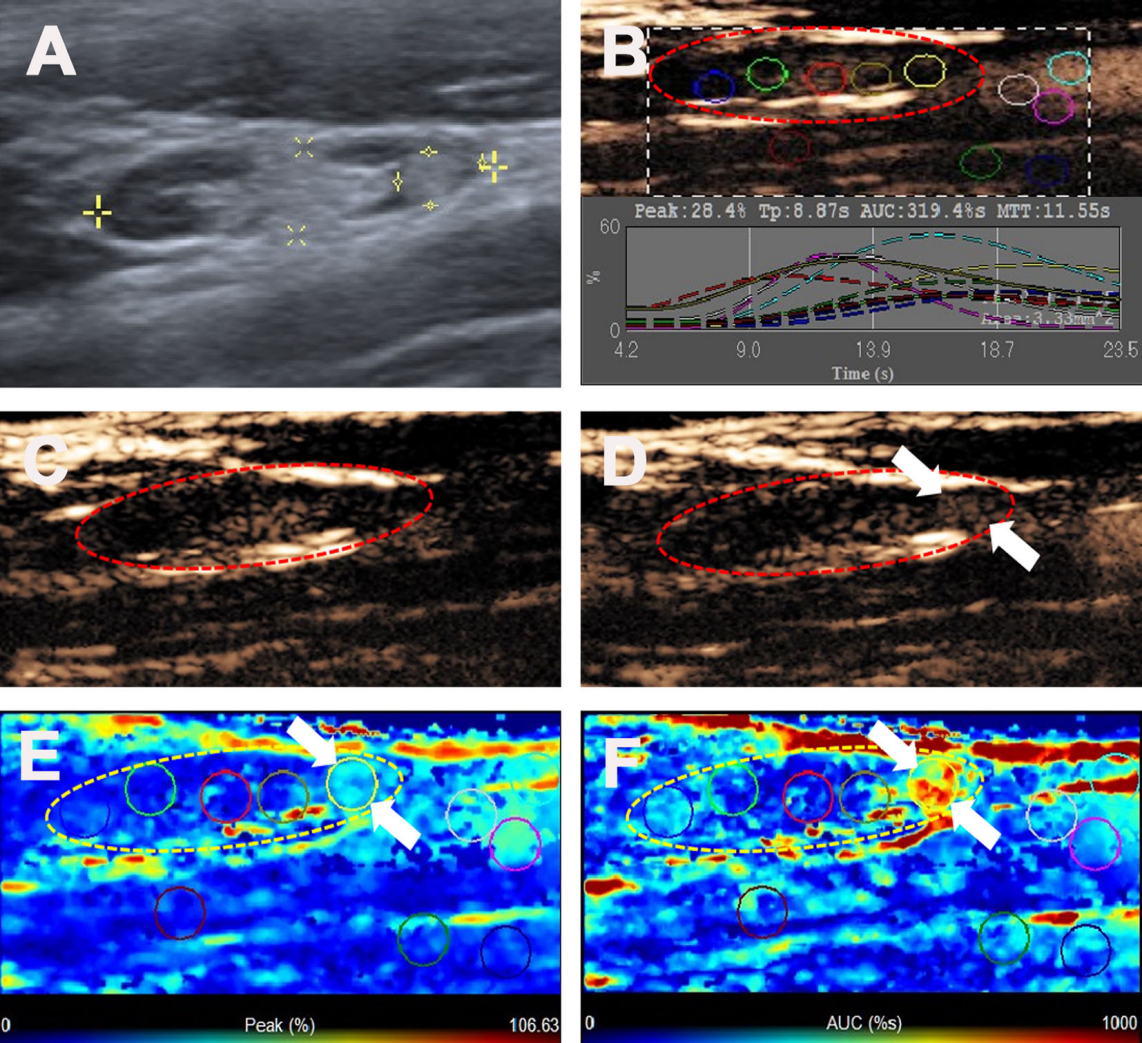
Metastatic lymph node in a 32-y-old male PTC patient. **(A)** The local metastatic foci with a hyper-echoic appearance in a LN (white cross) identified on conventional US. **(B)** Numeric values of peak, time to peak, mean transit time and AUC were automatically calculated based on the time-intensity curve. **(C)** CEUS image before injection of contrast-enhanced agent. **(D)** CEUS image 10 s after injection of contrast-enhanced agent, the local hyper-echoic appearance of LN showed hyper-enhancement. **(E)** Parametric color map indicating the PI values for the local metastatic foci of LN was higher than that of the other part of LN, equal to that of internal jugular vein and higher than that of the surrounding adipose tissue. **(F)** Parametric color map indicating the AUC values for the local metastatic foci of LN was higher than that of the other part of LN, equal to that of internal jugular vein and higher than that of the surrounding adipose tissue.

**Figure 4:**
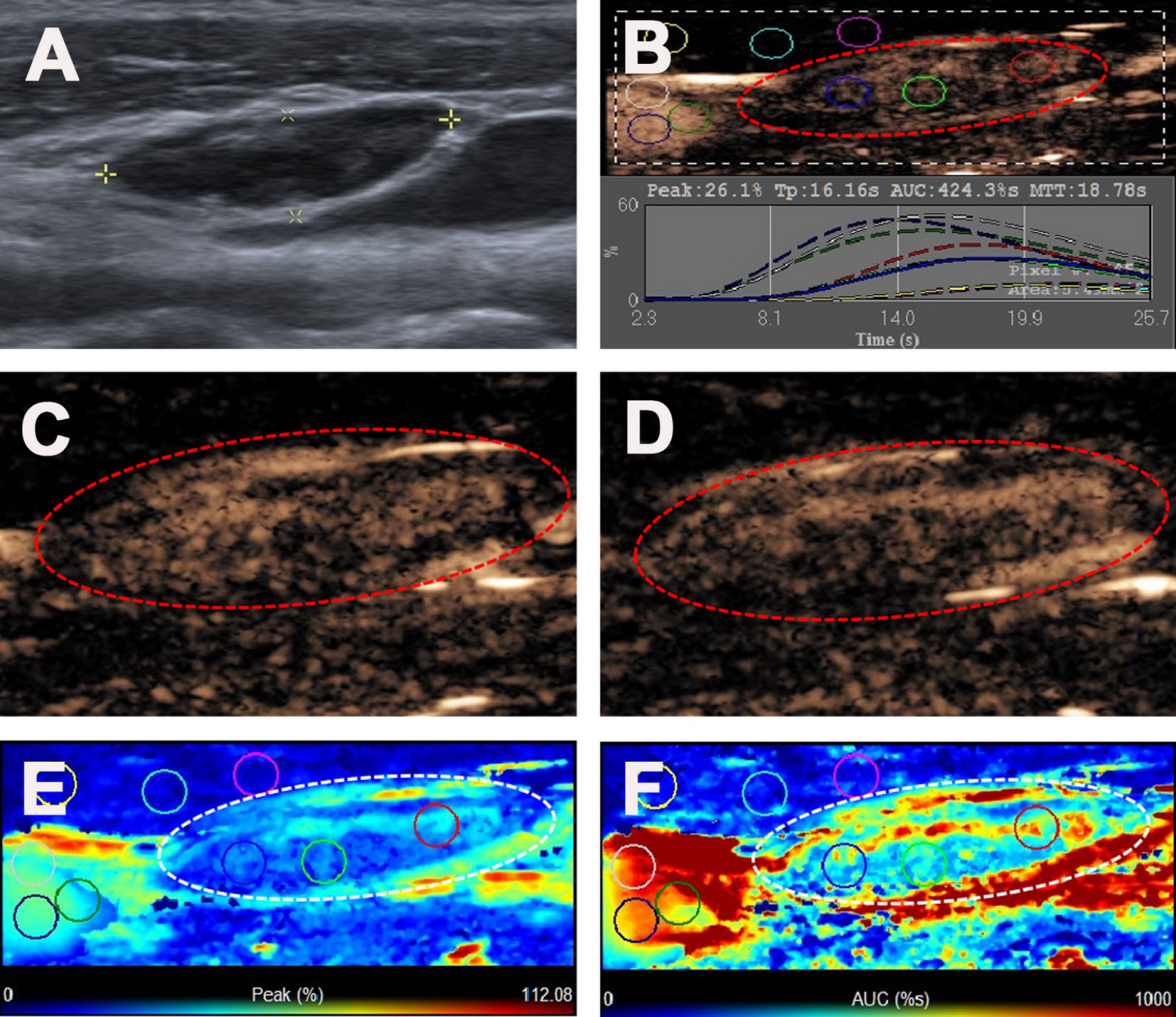
Reactive lymph node in a 35-y-old female PTC patient. **(A)** The LN with the absence of fatty hilum (white cross) identified on conventional US. **(B)** Numeric values of peak, time to peak, mean transit time and AUC were automatically calculated based on the time-intensity curve. **(C)** CEUS image 11 s after injection of contrast-enhanced agent, the LN enhanced from the center to the periphery. **(D)** CEUS image 18 s after injection of contrast-enhanced agent, the LN showed iso-enhancement. **(E)** Parametric color map indicating the PI values for the center of LN was higher than that of the periphery of LN, lower than that of internal jugular vein and higher than that of the surrounding adipose tissue. **(F)** Parametric color map indicating the AUC values for the center of LN was higher than that of the periphery of LN, lower than that of internal jugular vein and higher than that of the surrounding adipose tissue.

The ultrasonographic features of the malignant and benign LNs in PTC patients are summarized in **Table 2**. The typical metastatic LN ultrasonographic performance is illustrated in **Figure 1**. For patients with multiple abnormal LNs, each lymph node was observed separately by multiple injections of contrast agent. The mean diameters of the malignant and benign LNs in PTC patients were 14.86 ± 7.17 mm (range: 5-51 mm) and 12.11 ± 5.16 mm (range: 5-25 mm), respectively, and the malignant LNs were larger than the benign lymph nodes in this study (p = 0.019). Five conventional ultrasound signs were of diagnostic value for malignant LNs from PTCs. L/S < 2 was shown in 44.6% of the malignant LNs but only in 27.3% of the benign LNs (p = 0.039). The fatty hilum was absent in 91.1% of the malignant LNs but only in 77.3% of the benign LNs (p = 0.013) (**Figures 2**–**4**). A hyper-echogenicity appearance was found in 73.9% of the malignant LNs but only 15.9% of the benign LNs (p = 0.000) (**Figures 2**, **3**). Calcification was present in 51.0% of the malignant LNs but only in 20.5% of the benign LNs (p = 0.000) (**Figure 2**). Peripheral vascularity was present in 33.1% of the malignant LNs but only in 2.3% of the benign LNs (p = 0.000). However, cystic change was present in 16.6% of the malignant LNs and 6.8% of the benign LNs (p = 0.104), and this difference was not significant. For CEUS parameters, six signs were of diagnostic value for malignant LNs from PTCs. Hyper-enhancement was found in 78.3% of the malignant LNs but only 22.7% of the benign LNs (p = 0.000) (**Figures 2**, **3**). Heterogeneous enhancement was showed in 72.0% of the malignant LNs but only 15.9% of the benign LNs (p = 0.000) (**Figure 3**). Centripetal perfusion was found in 86.6% of the malignant LNs but only 11.4% of the benign LNs (p = 0.000). Ring enhancement was present in 60.9% of the malignant LNs but only 18.9% of the benign LNs (p = 0.000). A PI index >1 was shown in 77.7% of the malignant LNs but only 25.0% of the benign LNs (p = 0.000) (**Figures 2**, **3**). An AUC index >1 was found in 61.8% of the malignant LNs but only 22.7% of the benign LNs (p = 0.000) (**Figures 2**, **3**).

**Table 2:** Ultrasound Characteristics of the malignant and benign lymph nodes in PTC patients.

Characteristics	Malignant LNs (n=157)	Benign LNs (n=44)	*P* Value
**Conventional US parameters**			
Size (mm)	14.86 ± 7.17	12.11 ± 5.16	0.019*
L/S			0.039*
≥2	87 (55.4)	32 (72.7)	
<2	70 (44.6)	12 (27.3)	
Fatty hilum			0.013*
Present	14 (8.9)	10 (22.7)	
Absent	143 (91.1)	34 (77.3)	
Hyper-echogenicity			0.000*
Present	116 (73.9)	7 (15.9)	
Absent	41 (26.1)	37 (84.1)	
Calcification			0.000*
Present	80 (51.0)	9 (20.5)	
Absent	77 (49.0)	35 (79.5)	
Cystic change			0.104
Present	26 (16.6)	3 (6.8)	
Absent	131 (83.4)	41 (93.2)	
Peripheral vascularity			0.000*
Present	52 (33.1)	1 (2.3)	
Absent	105 (66.9)	43 (97.8)	
**CEUS parameters**			
Enhancement type			0.000*
Hypo- or iso-	34 (21.7)	34 (77.3)	
Hyper-	123 (78.3)	10 (22.7)	
Enhancement homogeneity			0.000*
Homogeneous	44 (28.0)	37 (84.1)	
Heterogeneous	113 (72.0)	7 (15.9)	
Perfusion			0.000*
Centripetal	136 (86.6)	5 (11.4)	
Centrifugal	21 (13.4)	39 (88.6)	
Ring enhancement			0.000*
Present	105 (60.9)	2 (18.9)	
Absent	52 (39.1)	42 (81.1)	
PI index			0.000*
>1	122 (77.7)	11 (25.0)	
≤1	35 (22.3)	33 (75.0)	
AUC index			0.000*
>1	97 (61.8)	10 (22.7)	
≤1	60 (38.2)	34 (77.3)	

PI, peak intensity; TP, time to peak; TP, time to peak time; AUC, area under the curve.

*p < 0.05 was considered a significant difference.

Binary logistic regression analysis was performed by including all of the statistically significant ultrasonographic variables (p < 0.05) from the univariate analysis in the model. The results indicated that hyper-enhancement (β= 1.182, odds ratio [OR] = 3.262, 95% confidence interval [CI] = 1.057-10.068, p = 0.040); centripetal perfusion (β= 3.340, odds ratio [OR] = 28.232, 95% confidence interval [CI] = 9.040-88.168, p = 0.000) and ring enhancement (β= 2.639, odds ratio [OR] = 14.006, 95% confidence interval [CI] = 2.715-72.248, p = 0.002) were independent characteristics related to malignant LNs for differentiating them from benign LNs (**Table 3**).

**Table 3:** Multivariate logistic regression analysis of ultrasonographic characteristics related to malignant lymph nodes distinguishing from benign lymph nodes.

Characteristics	Partial regression coefficient, β	Odds ratio	95% Confidence interval	*P* Value
Hyper-enhancement	1.182	3.262	1.057-10.068	0.040*
Centripetal perfusion	3.340	28.232	9.040-88.168	0.000*
Ring enhancement	2.639	14.006	2.715-72.248	0.002*

*p < 0.05 was considered a significant difference.

## Discussion

High-resolution US is a strongly recommended tool for the preoperative diagnosis of PTC and the evaluation of cervical LNs according to ATA guidelines. Some studies have reported that the CEUS features of PTCs could preoperatively predict the cervical LNs metastasis (14). Zhan et al. found that iso- or hyper-enhancement at peak time could predict cervical LNs metastasis in PTC patients (15). Tao et al. reported that peak of the nodule interior of 28.3750 or greater, and AUC of the peripheral ring of less than 3.2500 were independent risk factors of cervical LNs metastasis in PTC patients (16). And other previous studies have directly studied the clinical application of CEUS in the differential diagnosis of benign and malignant LNs. Nie et al. pointed out that CEUS examination was efficient in the differential diagnosis between head and neck lymphoma and malignant metastatic LNs (17). The findings of Cui et al. showed that CEUS with TIC analysis had potential diagnostic value in distinguishing tuberculous LNs from metastatic LNs (18). In line with this, Ling et al. demonstrated the good diagnostic value of qualitative and quantitative CEUS parameters in differentiating cervical benign LNs from malignant LNs in nasopharyngeal carcinoma patients (11). However, the cancerous LNs in these studies are not from PTC, thus the CEUS features of malignant LNs from other primary tumors are inconsistent with those of PTC. Hong et al. found that metastatic LNs more often manifested centripetal or asynchronous perfusion, hyper-enhancement, heterogeneous enhancement, perfusion defects and ring-enhancing margins than benign LNs on preoperative CEUS (13). Furthermore, Chen et al. showed that the characteristics of cervical LN metastasis from PTC on CEUS include centripetal perfusion, peripheral or mixed enhancement, and an enlarged range on CEUS compared with US (12). However, the inclusion criteria of metastatic and benign LNs were not all based on surgical pathology, and core needle biopsy or FNA were also included in these studies, which resulted in some inevitable false negatives. In our study, the histopathological results after surgery were used as the only reference standard for the final diagnosis of malignant and benign LNs to avoid unnecessary false negatives. In addition, the capability of CEUS both qualitative and quantitative for identifying metastatic cervical LNs from PTC was investigated. Our results suggest that, based on the imaging pattern and quantitative analysis, CEUS may hopefully be an excellent imaging technique for differentiation of malignant metastatic LNs and benign reactive LNs in PTC patients.

In this study, we first linked the hyper-echoic appearance of metastatic foci on conventional US with the hyper-enhancement on CEUS together. As shown in **Figures 1** and **3**, the microbubbles first entered the hyper-echoic appearance and showed local hyper-enhancement, indicating that the metastatic foci invaded the normal LN structure and possessed abundant blood vessels, which is consistent with the study of Wei et al. (19). In his study, the status of LN metastasis was further divided into four grades. Grade III and IV had a high-echoic appearance and showed hyper-enhancement on intravenous CEUS *via* intravenous injection and hypo-enhancement on lymphatic CEUS through superficial thyroid parenchyma injection.

According to our research, heterogeneous enhancement was observed in the majority of malignant LNs on CEUS, whereas benign LNs mostly manifested as homogeneous enhancement. Immature neovascularization and avascular necrotic areas are common in metastatic LNs, and they impede the distribution of contrast agent to these areas resulting in perfusion defects. However, in most reactive LNs, microbubbles flow easily from the fatty hilum and distribute to the whole LN rapidly to show homogeneous enhancement. This result was consistent with previous studies on the metastatic LNs from PTC (12, 13, 19).

For the CEUS perfusion pattern in our study, most metastatic LNs had a centripetal perfusion pattern, whereas most benign LNs had a centrifugal perfusion pattern. In benign reactive LNs, vessels enter the LN through the hilum and then spread in branches, which are characterized by microbubbles with enhancement from the center to the periphery presenting as centrifugal enhancement on CEUS. In contrast, when metastatic foci occur in LNs, tumor cells first invade the input lymphatic vessels and then subcapsular sinuses and locally induce neovascularization. The microbubbles spread from the periphery to the inner LN area presenting as centripetal enhancement on CEUS, which could present as peripheral vascularity on CDFI in metastatic LNs (12, 20). However, only a small portion (33.1%) of metastatic LNs showed peripheral vascularity on CDFI, compared with the majority (86.6%) of metastatic LNs, which exhibited centripetal enhancement on CEUS due to the higher sensitivity and accuracy of CEUS in the detection of structures with low-volume fluid flow (13, 21).

Furthermore, our study identified ring enhancement as one of the independent factors for differentiating metastatic LNs from reactive LNs in PTC patients. This CEUS feature is different from that of primary malignant thyroid tumors, and ring enhancement in the thyroid often indicates benign thyroid nodes (22, 23). The ring enhancement is likely due to metastatic foci entering lymph nodes through afferent lymphatic vessels and spreading from the marginal sinuses, which may block the draining lymphatic channels; thus, the periphery of the LN may be the last invaded and show a ring enhancement on CEUS (17, 20, 24).

Our previous studies quantitatively analyzed the CEUS parameters of thyroid nodes and provided some important information for identifying malignant thyroid nodes, such as PI and AUC (9, 10, 25). In this study, we used the PI index and AUC index to quantitatively evaluate the PI (mean maximum dose of microbubbles filling the ROI within a certain period of time) and AUC (mean total dose of microbubbles filling the ROI within a certain period of time), compared with those of adjacent tissue. We found that the majority of metastatic LNs from PTC showed PI > 1 and AUC >1, which was consistent with the above observation of hyper-enhancement of qualitative CEUS parameters. In our study, we compared the intensities of PI and AUC with both surrounding jugular vein and adipose tissue. This was different from other previous studies (11, 17), which compared these quantitative CEUS parameters of the whole LN with those of the surrounding adipose tissue. Thus, we compared the qualitative and quantitative CEUS parameters of LNs more objectively and accurately, which may provide valuable information for the evaluation of LNs in PTC patients.

The present study has several limitations. First, only reactive LNs were included in the benign group. Other benign lymphadenopathies, such as tuberculosis, purulent lymphadenitis, and granulomatous infections, were not investigated in this study. Second, unavoidable selection bias existed, and the malignant and benign LNs included in this study were confirmed by pathologic examination, whereas the other LNs that did not undergo surgery were not included. The lymph nodes diagnosed as benign by ultrasonography would be excluded in this article due to no evidence for surgery, limiting the practicality. Determination of suspicious LNs was dependent on obtained from this examination depend on the subjective evaluation of the clinician. Third, the number of cases included in this study was limited, especially in the benign group. The results of the present study necessitate a large-scale study with a prospective design to clarify these findings in the future.

## Conclusions

We provided the various sonographic characteristics of metastatic LNs from PTC patients, especially with respect to their differentiation from reactive LNs in PTC patients, and we evaluated the conventional US and CEUS findings. On preoperative US and CEUS, malignant LNs more frequently had L/S < 2, absence of a fatty hilum, presence of hyper-echogenicity, presence of calcification, peripheral vascularity, hyper-enhancement, heterogeneous enhancement, centripetal perfusion, ring enhancement, PI index > 1 and AUC index > 1 than benign LNs. Binary logistic regression analysis demonstrated that hyper-enhancement, centripetal perfusion, and ring enhancement are independent CEUS characteristics related to malignant LNs for their differentiation from benign LNs. Preoperative CEUS characteristics may serve as a useful tool to identify malignant cervical LNs from benign cervical LNs, which would help patients avoid unnecessary lymph node dissection or FNA.

## Data Availability Statement

The original contributions presented in the study are included in the article/supplementary material. Further inquiries can be directed to the corresponding author.

## Ethics Statement

The studies involving human participants were reviewed and approved by the Ethics Committee of Second Xiangya Hospital, Central South University, China. The patients/participants provided their written informed consent to participate in this study.

## Author Contributions

CN contributed to the conception and design of the work. FF and YG participated to data analysis and manuscript writing. LL, FY, ZZ, XL, QZ, KT, YX, RZ, and SC participated to data collection and patients’ follow-up. All authors contributed to the article and approved the submitted version.

## Funding

This project was funded by the National Natural Science Foundation of China (81974267) and the Science and Technology Innovation Program of Hunan Province (2021RC3033).

## Conflict of Interest

The authors declare that the research was conducted in the absence of any commercial or financial relationships that could be construed as a potential conflict of interest.

## Publisher’s Note

All claims expressed in this article are solely those of the authors and do not necessarily represent those of their affiliated organizations, or those of the publisher, the editors and the reviewers. Any product that may be evaluated in this article, or claim that may be made by its manufacturer, is not guaranteed or endorsed by the publisher.
